# The Ebola Data Platform: A prospective, standardised, clinical dataset collected during the 2013-2016 West African Ebola outbreak

**DOI:** 10.12688/wellcomeopenres.22483.1

**Published:** 2024-09-26

**Authors:** Laura Merson, Trokon Omarley Yeabah, Samantha Strudwick, Tamba Fayiah, Jennifer H. Lee, Musa Martin Feika, Gemma Buck, Kwame Oneill, Kalynn Kennon, Mahamoud Sama Cherif

**Affiliations:** 1 ISARIC, Pandemic Sciences Institute, University of Oxford, Oxford, England, UK; 2 National Public Health Institute Liberia, Monrovia, Liberia; 3 Infectious Diseases Data Observatory (IDDO), University of Oxford, Oxford, England, UK; 4 Partnership for Research on Vaccines and Infectious Diseases in Liberia, Monrovia, Liberia; 5 Directorate of Health Security and Emergency, Ministry of Health and Sanitation, Freetown, Sierra Leone; 6 College of Medicine and Allied Health Sciences, University of Sierra Leone, Freetown, Sierra Leone; 7 Faculty of Sciences and Health Technics, Gamal Abdel Nasser University of Conakry, Conakry, Guinea; 8 Direction Regionale de la Santé de Faranah, Ministère de la santé et de l'hygiène publique, Faranah, Guinea

**Keywords:** Ebola virus disease, data, data sharing, outbreak, viral haemorrhagic fever

## Abstract

The Ebola Data Platform (EDP) was developed to strengthen knowledge and capacity across health, research, and humanitarian communities to reduce the impact of Ebola through responsible data use. This collaborative initiative was established by West African governments, NGOs, academic organisations, and intra-governmental health organisations directly involved in the 2013–2016 West African Ebola outbreak. The platform was established to provide a centralised, standardised dataset of individual patient data collected during the outbreak for the purpose of research to improve Ebola treatment and control, and includes over 13,600 patient records of individuals infected and treated from 22 different Ebola treatment centres across Guinea, Sierra Leone, Liberia, and Nigeria. Patient data are available from treatment centre triage and admission, inpatient clinical observations, and outcomes, with outpatient follow-up available for some datasets. Data include signs and symptoms, pre-existing comorbidities, vital signs, laboratory testing, treatments, complications, dates of admission and discharge, mortality, viral strains, and other data. This publication describes characteristics of the EDP dataset, its architecture, methods for data access and tools for utilising the dataset.

## Introduction

Since the Ebola virus disease was first identified in 1976, nearly 35,000 infections have been recorded. More than 90% of these infections have occurred in the last ten years ^1, 2^. Despite the volume of Ebola infections reported in recent outbreaks, Ebola virus disease research has been heavily constrained by the lack of accessible and standardised data, with research outputs restricted to analyses of small data sets from isolated sources.

The urgency and demands of outbreak management, the resource limitations of the affected countries, and the perceived sensitivities of sharing data have repeatedly inhibited the preservation and utilisation of data collected. This has resulted in inadequate empirical and scientific evidence to inform advances in diagnosis, triage, management, and follow-up of suspected and confirmed Ebola patients ^3^. Furthermore, the West African Ebola virus disease outbreak that took place from 2013 to 2016, rapidly spread across borders and highlighted the urgent need for stronger response capacity and collaboration among affected countries. In response to these needs, the Ebola Data Platform project was established with the aim of building a shared, centralised repository of harmonised data that would enable ethical and equitable access to existing Ebola data for use by scientific, health, research, and public health communities. Enabling the conduct of more robust analyses of pooled data could help reduce the impact of Ebola by answering critical questions to improve future outbreak response and patient care.

**Figure 1.**
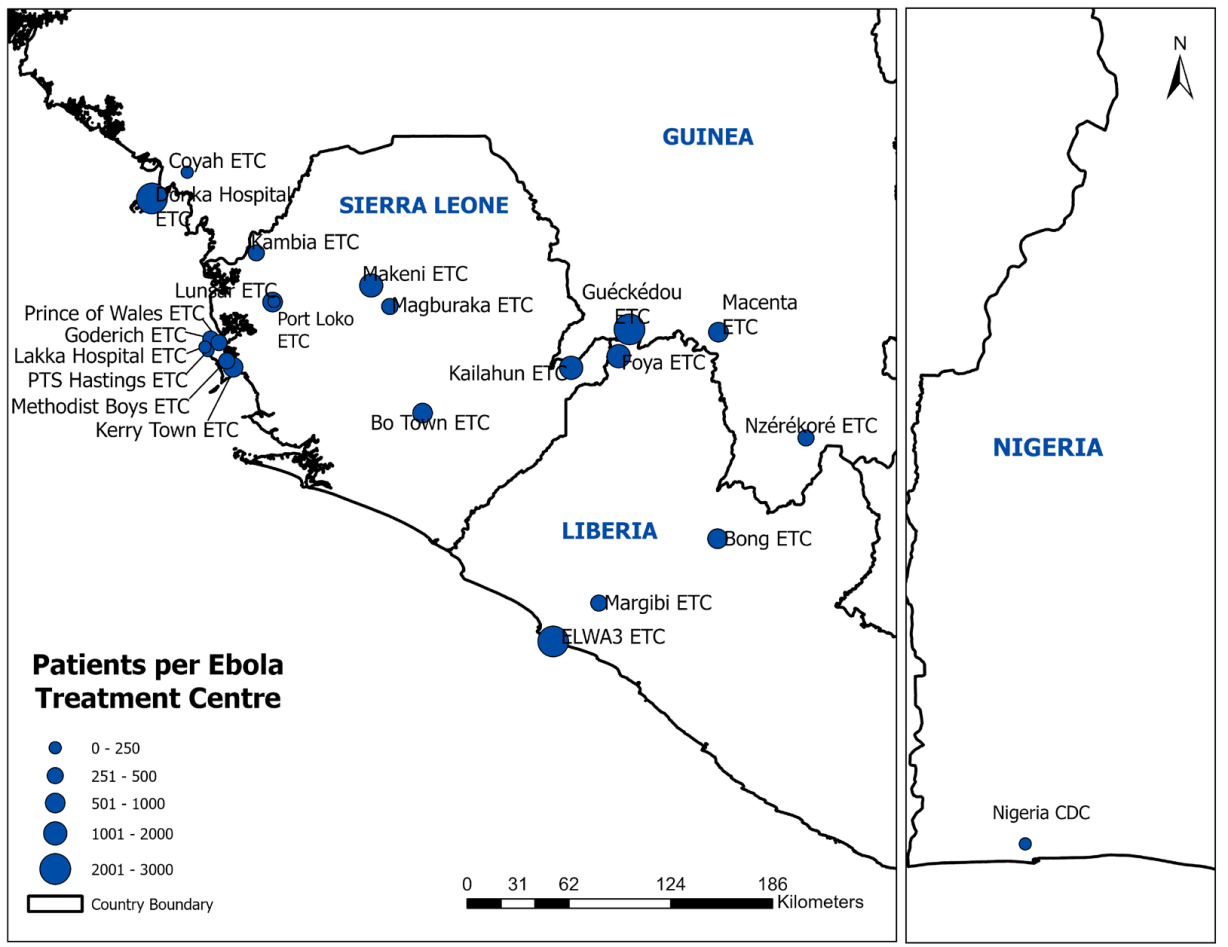
Ebola Treatment Units that contributed data to the Ebola Data Platform database.

The Ebola Data Platform (EDP) project is the initiative and result of a multi-disciplinary collaboration between the national health agencies of Guinea, Liberia, Sierra Leone, and Nigeria; the humanitarian organisations Médecins Sans Frontières and International Medical Corps; academic groups at the University of Oxford, the West African Consortium, and the West African Taskforce for Emerging and Re-emerging Diseases; the charitable foundation Wellcome; and the public health actors at the West African Health Organization and the World Health Organization. The platform currently hosts the individual patient data from 14,191 patient records from admission to 22 Ebola treatment centres across 4 countries ( Figure 1). When accounting for related records across multiple datasets to the best ability with anonymised data, it is estimated these records account for over 13,671 unique patients. All contributed data have been standardised to a uniform format and are available to researchers through a governed data access mechanism.

The EDP is the world’s first global data repository for clinical, epidemiological, and laboratory data on Ebola virus disease, aggregating and harmonising millions of data points from thousands of individual patient records collected during the West African Ebola outbreak. In addition to creating this standardised dataset, a key element of the EDP’s mission is to enable the use of the platform to strengthen and promote research by researchers in Ebola-affected countries through collaboration, training and capacity sharing.

## Methods

### Data collection

The EDP was established to aggregate and standardise disparate datasets from the many organisations that collected individual patient-level data as a part of the care provided in Ebola treatment centres. As a key pillar of the public health response, the centres delivered and captured data on clinical care and follow-up, laboratory services, epidemiological investigation, and observational research. Clinical trials were conducted at some centres. Data were collected on either paper or electronic forms using variables selected by the organisation managing the respective clinical treatment centres and studies. The data were submitted to the EDP by the organisation responsible for primary data collection under the authority of the responsible ministry of health or public health agency.

### Engagement, consent, and ethics

To ensure robust and representative governance for the platform, all members of the research, health and humanitarian communities were invited to participate in the collaborative design of the platform’s governance framework ( https://www.iddo.org/ebola/governance/structure). Representatives of the health authorities of the four most affected countries during the 2013–2016 outbreak, worked together with international public health agencies, researchers, funders and NGOs, to ensure that data hosting and access met the appropriate legal, ethical and scientific requirements. 

Most data on the platform were collected in the context of a public health emergency response. Informed consent was not sought to collect or use data because recording of clinical status and laboratory results was a part of routine care and public health measures and not for the purpose of research. A limited volume of data on the platform were collected as a part of an observational or interventional research study, in which case informed consent was obtained for data to be collected as a part of the research study in question. In some cases, approval to share data with secondary researchers for the purpose of future analyses was included in the signed consent form.

The EDP facilitates the use of de-identified patient data for the purpose of research analysis, in most cases, without specific patient consent. Due to the challenges of seeking retrospective consent for use of data within this setting, the responsible ethics committees were asked to authorise a waiver of consent in the sharing of the datasets via the platform. This approach was decided following a review of international guidelines, evaluation of contextual considerations, the design of comprehensive safeguards to protect the rights and interests of patients and communities, and the implementation of a benefit sharing framework.

Data hosting and access according to the EDP security, privacy and governance frameworks were approved by the national ethics committee in each contributing country and by the Oxford Tropical Research Ethics Committee:

- Guinea National Committee for Health Research Ethics (Comite National D’Ethique pour la Recherche en Sante; approval number: 104/CNERS/18; date of approval: 19 October 2018)
- Sierra Leone Ethics and Scientific Review Committee (no reference number; date of approval: 6 November 2018)
- Liberia National Research Ethics Board (approval number: NREB-016-19; date of approval: 11 July 2019)
- National Health Research Ethics Committee of Nigeria (approval number: NHREC/01/01/2007; date of approval: 8 January 2020)
- Oxford Tropical Research Ethics Committee (approval number: 515-18; date of approval: 28 March 2018)

### Data standardisation

There was substantial heterogeneity in the raw datasets contributed to the EDP with variations in the information collected, study design, data collection methods, units, language, data formats and outcome measures. To standardise the disparate datasets, a process of curation was applied to fit the data to a unified data model. The following sections outline Infectious Diseases Data Observatory (IDDO)’s systems for the implementation of data curation as previously described in the COVID-19 dataset publication ^4^.

***Establishing a common data model.*** In the absence of an existing universal standard for Ebola data at the commencement of the project, we partnered with the Clinical Data Interchange Standards Consortium (CDISC) to develop a well-documented data model to accommodate the range of data required for outbreak response. The CDISC Ebola Therapeutic Area User Guide ^5^ describes the most common biomedical concepts relevant to patient data on Ebola virus disease. It includes the metadata needed to represent these data, consistent with controlled terminologies and the CDISC models for data collection and tabulation, specifically, Clinical Data Acquisition Standards Harmonisation (CDASH) and Study Data Tabulation Model (SDTM) ^5^. Use of these models for the EDP data enabled robust standardisation of the disparate datasets included. Further advantages of these models include the adaptation to any number of events and the capture of unique variables that were collected for each patient so that accurate denominators can be calculated.

***De-identification.*** Data uploaded to the Ebola Data Platform are manually reviewed to ensure no direct identifiers are included. Direct identifiers, including those listed in the UK General Data Protection Regulation ^6^ and the US Health Insurance Portability and Accountability Act ^7^ are permanently deleted before data are curated through the processes below.

***Pre-mapping.*** Data and all documentation shared with the data, such as dictionaries, protocols, publications, and data collection forms, are reviewed by the data curator to fully understand the contents of the dataset. Queries are raised with the data contributor when required. Each variable in the dataset is assigned to the appropriate SDTM domain(s), variable(s), and controlled vocabulary (if applicable) according to the rules found within the IDDO SDTM Implementation Manual ( https://www.iddo.org/tools-and-resources/data-tools). The implementation manual chronicles each type of data curated to the platform and is consulted and updated with each new dataset to ensure consistency across the repository. An audit trail of the assignments is also recorded in a dataset-specific SDTM mapping guide.

***Data wrangling.*** For formatting and coding, the contributed datasets are loaded into Trifacta®, a data wrangling programme. Transformations can include merging files, splitting variables into separate domains, applying controlled terminology to variables, and adding created variables as required. Defined standardization, conversion, and categorization formulas are also used as described in the IDDO SDTM Implementation Manual. Transformations on the contributed data (in the interests of standardization) are recorded and stored in a form that documents the transformation and enables it to be reproduced.

***Review and edit checks.*** Data are then run through Pinnacle 21c® (community version) software, a CDISC standards compliance-verification tool that checks the standard SDTM implementation guide rules and requirements for regulatory submission. The resulting checks and warnings are assessed for applicability to the individual dataset. The data are also run through standard edit checks to identify possible mapping errors separate from SDTM conformance. The curator adjusts the mapping as needed to make corrections.

***Privacy assurance.*** A quantitative assessment of disclosure risk is executed on all data approved for access by external researchers. Based on the results of the assessment, data are bucketed, redacted, or masked as required to ensure that the maximum probability of re-identification across all records is below the conservative risk threshold of 0.09 as stated in the European Medicines Agency (EMA) policy for the public disclosure of the clinical reports ^8^.

Original subject identification codes are replaced with randomly generated, unique subject identifiers each time a dataset is shared with researchers. This reduces the risk of data being linked to other data outside of the platform.

**Figure 2.**
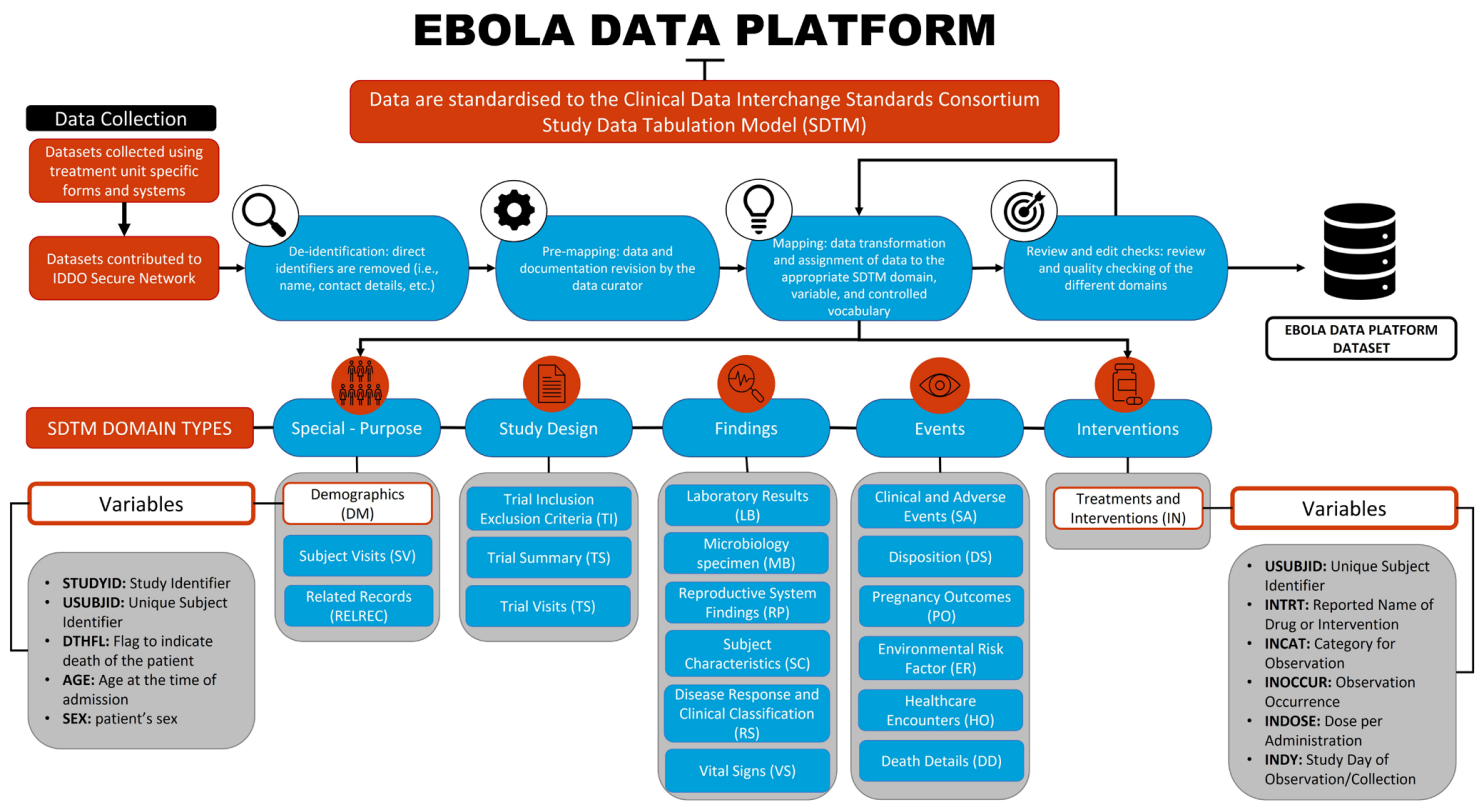
Overview of the Ebola Data Platform database.

Figure 2 describes the workflow from data acquisition to the final, pooled dataset that researchers can access to conduct their research.

***Validation.*** Data uploaded to the IDDO EDP are verified during the ‘pre-mapping’ and ‘data review and edit checks’ processes described above. Interpretation of the data dictionary and any missing values are queried directly with staff at the organisation responsible for data collection where possible.

Due to the challenges of collecting data in Ebola treatment units (ETUs), which were often overcrowded, during a public health emergency, data quality is highly variable across the dataset. Where queries could not be resolved, inconsistent data have been retained so that decisions on cleaning and exclusions may be decided by researchers using the data, according to the individual analyses.

Note that data are described according to their status on 30 May 2023. Additional curation processes may be applied to the data as IDDO evolves methods in data processing. 

### FAIR data

The EDP follows the FAIR ^9^ data principles to make the data it hosts more Findable, Accessible, Interoperable and Reusable. A digital object identifier has been created for each contributed dataset, enabling persistent data provenance and information on the data source, licence and access mechanism ^10– 24^. A list of each dataset held in the EDP can be found in the data inventory ( https://www.iddo.org/ebola/data-sharing/accessing-data) with details on the contributing organisation, location of the treatment centre, dates of data collection, number of patients by demographic group, and types of data included.

## Data records

The EDP dataset is available from the Infectious Diseases Data Observatory – IDDO at https://doi.org/10.48688/cpwp-ft84. The Ebola dataset is a relational database consisting of 19 tables; 16 patient-level tables and three dataset-level tables, each representing a domain of information set out in the CDISC SDTM data model. Unique identifiers link these with the suffix ‘ID.’ For example, USUBJID refers to the subject's unique identifier, which is the primary key for assessing individual-level data; STUDYID contains the unique identifier for an individual hospital or network of hospitals. Each table defines and tracks different aspects of illness and treatment.

### Data tables

The patient-level tables (i.e., domains) included in the dataset are Demographics (DM), Disposition (DS), Death Details (DD), Environmental Risk (ER), Healthcare Encounters (HO), Treatments and Interventions (IN), Laboratory Results (LB), Microbiology Specimen (MB), Pregnancy Outcomes (PO), Related Records (RELREC), Reproductive System Findings (RP), Disease Response and Clinical Classification (RS), Clinical and Adverse Events (SA), Subject Characteristics (SC), Subject Visits (SV) and Vital Signs (VS). These tables include a unique subject identifier (USUBJID) that relates the information of a single patient distributed across the multiple tables.

**Figure 3.**
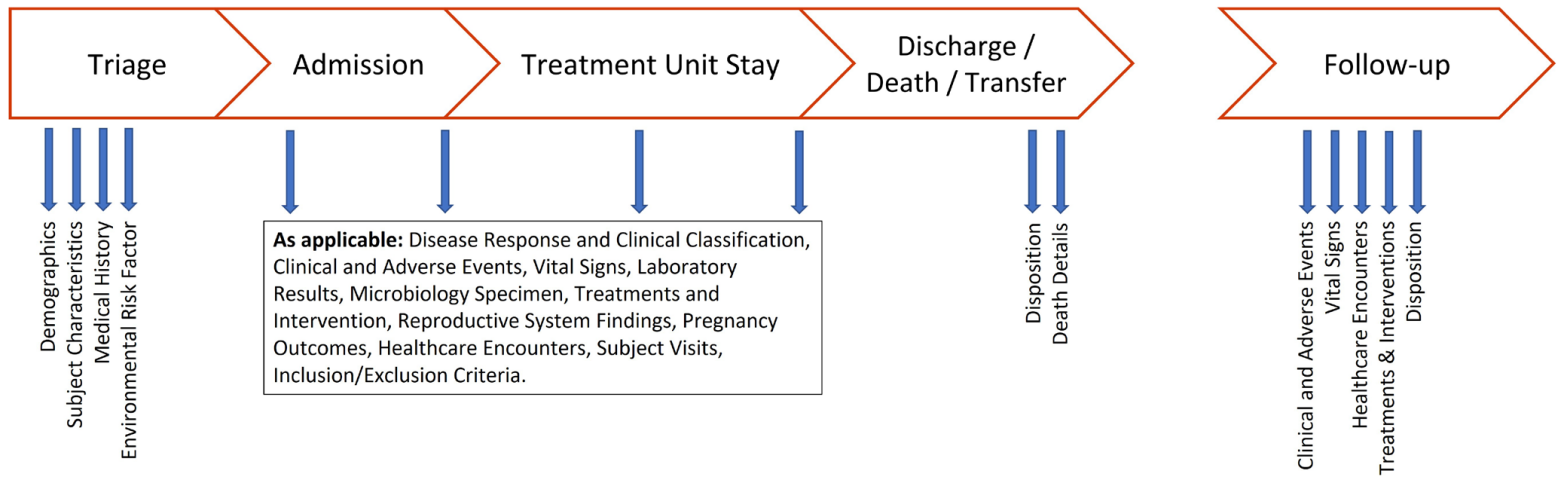
Data collection points for each data type.

The Trial Summary (TS), Trial Inclusion Exclusion Criteria (TI), and Trial Visits (TV) are dataset-level domains. These domains have information about the uniqueness of each dataset, such as where they were collected, the selection criteria for the individuals included, the clinical trial study design, and the research study visit schedule. They do not include individual patient-level data but can be linked to individual data on each patient-level table by the study or dataset identifier (STUDYID). Figure 3 illustrates the data collection times for each data type. Table 1 describes how data are distributed across the domain data tables and how many unique patients are included in each table.

**Table 1.** Patient numbers and variables per domain.

Domain	Domain Name	Variables	Rows	Patients	Description
Patient-level domains					
**DD**	Death Details	12	837	725	Information related to deaths of patients
**DM**	Demographics	16	14191	13671	Essential standard, non-clinical information that describe an individual
**DS**	Disposition	18	14318	14053	Medical status or outcomes
**ER**	Environmental Risk	25	40187	8333	Assessments of potential exposures to or risk factors associated with the disease
**HO**	Healthcare Encounters	21	34355	12689	Inpatient and outpatient healthcare events
**IN**	Treatments and Interventions	40	1619418	6391	Experimental, concomitant, and prior medications and treatments
**LB**	Laboratory Results	24	18066	744	Laboratory test data (except microbiology)
**MB**	Microbiology Specimen	29	46532	12778	Detection, identification, and quantification of microorganisms
**PO**	Pregnancy Outcomes	10	62	31	The outcome of pregnancy; pre-term, live birth
**RELREC**	Related Records	4	1050	530	Related patient IDs for patients appearing in more than one dataset
**RP**	Reproductive System Findings	20	4414	3666	Reproduction-related information
**RS**	Disease Response and Clinical Classification	18	14062	2146	Clinical classifications based on published testing criteria
**SA**	Clinical and Adverse Events	32	3069933	13586	Clinical events of interest
**SC**	Subject Characteristics	11	65297	12679	Non-clinical information that describe an individual
**SV**	Subject Visits	6	2904	2903	Case report form dates
**VS**	Vital Signs	28	106963	6317	Measurements of the body's essential functions are monitored during visits or hospitalisation
Dataset-level domains					
**TS**	Trial Summary	11	970	NA	Study level domain. Variables that describe the dataset
**TI**	Trial Inclusion Exclusion Criteria	5	52	NA	Study level domain. Trial-level inclusion and exclusion criteria
**TV**	Trial Visits	8	101	NA	Study level domain. Trial-level planned visits

When shared through the governed data access mechanism, the Ebola database is provided as a collection of comma-separated value (CSV) files (i.e., tables). Notably, where data transformations are made during the database construction process, care is taken not to modify raw study data. The teams performing analyses can develop analytic codes based on assumptions they deem appropriate.

### Patient characteristics

Of the 13,671 individual patients, 6,979 (51.0%) were male, 6,462 (47.3%) were female and the sex was unknown for the remaining 230 (1.7%). The majority of patients (n=8,154, 59.6%) were aged 16-45 years. The number of individual patients by ETU country include 5,100 (37.3%) patients from Sierra Leone, 4,928 (36.0%) patients from Guinea, 3,624 (26.5%) patients from Liberia and 19 (0.1%) patients from Nigeria ( Table 2).

**Table 2.** Ebola Data Platform patient population.

	N (% of 13671 patients)
**Sex**	
Male	6979 (51.0%)
Female	6462 (47.3%)
Unknown	230 (1.7%)
**Age**	
0–5	1246 (9.1%)
6–15	1505 (11.0%)
16–45	8154 (59.6%)
46+	2433 (17.8%)
Unknown	333 (2.4%)
**Country**	
Guinea	4928 (36.0%)
Liberia	3624 (26.5%)
Nigeria	19 (0.1%)
Sierra Leone	5100 (37.3%)
**Laboratory ** **confirmed ** **infections**	
EVD only	7339 (53.7%)
EVD and malaria	578 (4.2%)
Malaria only	324 (2.4%)
Missing	5430 (39.7%)

Of the 7,909 patients with laboratory confirmed EVD, death was reported in 3,162 (40.0%) patients, recovery in 3,598 (45.5%), with the outcome unknown in 1,146 (14.5%) patients. Pregnancy was recorded for 191 (5.3%) of the 3,619 female patients with laboratory confirmed EVD. Lethargy/fatigue was the most reported sign and symptom (n=9,113, 83.1%), followed by fever (n=8,528, 80.4%), anorexia (n=7,701, 74.0%) and headache (n=7,182, 67.4%) ( Table 3). Data of convalescent patients from clinic visits post ETU discharge are available for 76/7909 (0.9%) EVD positive patients.

**Table 3.** Outcomes and clinical features of laboratory confirmed EVD patients.

Outcome	n (% of 7909 patients)
Death	3162 (40.0%)
Recovery	3598 (45.5%)
Unknown	1146 (14.5%)
Missing	3 (0.0%)
Pregnancy	n (% of 3619 female patients)
Pregnant Indicator	191 (5.3%)
Missing	3428 (94.7%)
Signs & symptoms	n/N with data (%)
Lethargy/Fatigue	9113/10963 (83.1%)
Fever	8528/10607 (80.4%)
Anorexia	7701/10407 (74.0%)
Headache	7182/10663 (67.4%)
Aching muscles or joints	792/3201 (24.7%)
Vomiting	6963/10811 (64.4%)
Diarrhoea	6456/10754 (60.0%)
Stomach pain	6369/10533 (60.5%)
Internal and external bleeding	2176/10186 (21.4%)
Difficulty swallowing/Sore throat	1532/5343 (28.7%)
Hiccups	1793/10209 (17.6%)
Difficulty breathing	2601/9383 (27.7%)
Impaired kidney or liver function	610/8681 (7.0%)
Rash	458/8368 (5.5%)

## Pioneering data science for emerging infections research

The EDP’s accomplishments and success are evidenced, and were driven, by the uptake and participation of several governments, NGOs, academic organisations and intra-governmental health organisations. The open and collaborative effort and shared ownership of this platform catalysed new directives in emerging infections data sharing and harmonisation.

With no existing universal standards for Ebola, the desire to harmonise disparate data sets to the most rigorous international standards led to the inaugural partnership between CDISC and use of the CDISC SDTM data model in the emerging infections community. This partnership extended beyond the data harmonisation of existing data as described above, also leading to the development of prospective data capture tools. In most emerging infections, as recognised in Ebola, data are often collected under extremely challenging conditions. To minimise future inconsistent, incongruent and ambiguous primary data capture, a case record form (CRF) of the suggested core clinical dataset for Ebola virus disease was collaboratively developed and annotated with CDASH variable names compliant with CDISC Standards. The resulting Ebola CRF can be accessed at https://www.iddo.org/document/isaric-who-ebola-infection-core-case-report-form-2014. This work paved the way for quality data collection and aggregation of data in emerging infections and has since been built on further by WHO, CDISC and other organisations as evidenced in more recent Ebola outbreaks and the COVID-19 pandemic ^25– 29^.

The EDP has successfully demonstrated that disparate data collected from multiple sites during an epidemic response can be standardised and harmonised for FAIR and equitable access for re-use. Furthermore, this project has demonstrated how existing data can be optimised by international researchers for subsequent analysis while collaboratively including and appropriately recognising the researchers responsible for primary data collection ^30, 31^. Thousands of patient-level data have been shared with the EDP and dozens of researchers in Ebola-affected countries have received new training in research, data management and statistical analysis. Novel solutions to historic barriers have enabled a data access model that has already positively impacted our preparedness for future pandemics and can further accelerate the understanding of emerging infections.

## Data Availability

The EDP dataset is available to researchers through a governed data access mechanism. The countries and organisations who contributed data to the EDP retain ownership and decision-making authority over their data. Researchers may request access to data on the EDP by submitting a data access application via https://www.iddo.org/ebola/data-sharing/accessing-data. Decisions on all applications are made by an independent Data Access Committee overseen by the World Health Organisation and TDR, the Special Programme for Research and Training in Tropical Diseases. Applications are reviewed for compliance with the EDP Data Access Guidelines of the EDP ( https://www.iddo.org/ebola/data-access-guidelines). All approved applications are publicly available ( https://www.iddo.org/ebola/research/approved-data-access-applications). The technical, governance and ethical framework for the data access process were collaboratively developed by the EDP Steering Committee ( https://www.iddo.org/ebola/governance/ebola-steering-committee). The framework is designed to promote access to data for research while protecting the rights and privacy of the people and communities from which the data originate and respecting the investment of the healthcare workers and researchers who conducted the studies and collected the data. In addition to criteria of scientific value and validity, applications for data access are reviewed for ethics and equity. This includes approval by the responsible ethics committee(s) and plans to ensure that the analysis brings value to Ebola-affected countries through evidence, collaboration and/or capacity strengthening. To optimise the utility of the dataset, a Research Agenda ( https://www.iddo.org/document/ebola-research-agenda-public-consultation-plateforme-de-donnees-ebola-programme-de) was developed by the EDP Steering Committee to promote the use of the EDP dataset to address research questions prioritised by Ebola-affected countries. This list was approved by the National Research Ethics Committees of the countries where data originate. Applications for data access may address the questions included on the research agenda or other relevant knowledge gaps. The EDP data have been used to generate new analyses and strengthen the evidence for the clinical management and treatment of Ebola. Published examples to date include a meta-analysis of selected studies to determine the suitability of existing data as a nonrandomized control group comparison for future clinical studies of experimental Ebola treatments, and use of the EDP dataset to inform a machine learning-derived prognostic model to predict clinical outcomes in children infected with Ebola virus ^30, 31^. EDP data have additionally been used for several Masters and PhD theses by students across the African continent. Details of training and engagement activities are available at https://www.iddo.org/ebola/research/training-engagement. Open access to all research outputs is available at https://www.iddo.org/ebola/research/approved-uses-platform-data. Recognition of the data source is pivotal to the success of the data-sharing movement in ensuring fair use of data ^9, 32– 35^. An important consideration for this project was implementing infrastructure to trace data provenance and promote direct citation of those who collect and share data with the EDP. Accordingly, every individual data set shared with the EDP has a Digital Object Identifier (DOI) minted via DataCite ^10– 24^. Publications and outputs of EDP data users are encouraged to cite all applicable dataset DOIs. As successfully demonstrated in Genisca *et al*., (2022) ^30^, the citation of DOIs increases the persistent findability of both the original and harmonised study data and documentation and ensures appropriate recognition of the data source. The code used to generate the tables and analysis included in this manuscript are available at https://github.com/toy2022/Ebola_paper/tree/main. Archived code: https://doi.org/10.5281/zenodo.13711747 ^36^ License: CC-BY 4.0
