# Supplementary material for: The Ebola Data Platform: A prospective, standardised, clinical dataset collected during the 2013-2016 West African Ebola outbreak
Source: Wellcome Open Res. 2024 Sep 26;9:548. [Version 1] doi: 10.12688/wellcomeopenres.22483.1 (PMC11499738; doi:10.12688/wellcomeopenres.22483.1)
Supplement: Peer review history

# Reviewer report: Reviewer response for version 1

**DOI:** 10.21956/wellcomeopenres.24772.r101920

**Published:** 2024-10-23

**Authors:** Jason Kindrachuk

**Affiliations:** 1 University of Manitoba, Winnipeg, Manitoba, Canada

The authors describe the Ebola Data Platform which provides a completely unique database for access to deidentified patient data from across Guinea, Liberia, Sierra Leone, and Nigeria. 

 In agreement with Reviewer 1, the EDP is an excellent resource and there are few comments. The comments provided by reviewer 1 are agreed upon and I would add the following minor recommendations in the presentation of the report:

**Minor comments:**

- Figure 1: Small critique here to try and ensure that ETC labels/designations are positioned to be readable (e.g. Lonka Hospital ETC). Alternatively, these could be provided as an added table beside the figure.
- A small suggestion to ensure consistency in the presentation of Ebola virus or Ebola virus disease rather than using "Ebola" only.
- Figure 1 has all sites listed as ETCs but ETU is used in the Results section of the manuscript. Please ensure consistency in the use of either ETC or ETU through the manuscript.
- Table 3: Would recommend listing these in descending order of either n or n/N values.

-

Are sufficient details of methods and materials provided to allow replication by others?

Yes

Is the rationale for creating the dataset(s) clearly described?

Yes

Are the datasets clearly presented in a useable and accessible format?

Yes

Are the protocols appropriate and is the work technically sound?

Yes

Reviewer Expertise:

My research focuses on emerging zoonotic infectious diseases including filoviruses and orthopoxviruses with a particular focus on West and Central Africa.

I confirm that I have read this submission and believe that I have an appropriate level of expertise to confirm that it is of an acceptable scientific standard.

# Reviewer report: Reviewer response for version 1

**DOI:** 10.21956/wellcomeopenres.24772.r101918

**Published:** 2024-10-18

**Authors:** Caroline Persson

**Affiliations:** 1 Denver Health & Hospital Authority, Denver, Colorado, USA

The article describes a novel and innovative approach to collating thousands of records across multiple partners. The partnership has the ability to inform and promote rich and robust information on Ebola-related medical records in an ethically approached manner.

 There are not many recommendations to elaborate on, one question below related to data shortcomings as could see this data set being highly accessed and requested to inform healthcare.

 1) Would recommend more clarity into the data shortcomings from the dataset. References shortfalls from disparate data, what should the data not be requested for?

Are sufficient details of methods and materials provided to allow replication by others?

Yes

Is the rationale for creating the dataset(s) clearly described?

Yes

Are the datasets clearly presented in a useable and accessible format?

Yes

Are the protocols appropriate and is the work technically sound?

Yes

Reviewer Expertise:

Special pathogens, infection prevention, and control and healthcare systems. Networks and collaboration within healthcare.

I confirm that I have read this submission and believe that I have an appropriate level of expertise to confirm that it is of an acceptable scientific standard.
